# Cancer phylogenetic inference using copy number alterations detected from DNA sequencing data

**DOI:** 10.1016/j.cpt.2024.04.003

**Published:** 2024-04-18

**Authors:** Bingxin Lu

**Affiliations:** a School of Biosciences and Medicine, University of Surrey, Guildford GU2 7XH, UK; b Surrey Institute for People-Centred Artificial Intelligence, University of Surrey, Guildford GU2 7XH, UK

**Keywords:** Clonal evolution, Phylogenetic inference, Somatic mutation, Copy number change, Chromosomal instability

## Abstract

Cancer is an evolutionary process involving the accumulation of diverse somatic mutations and clonal evolution over time. Phylogenetic inference from samples obtained from an individual patient offers a powerful approach to unraveling the intricate evolutionary history of cancer and provides insights that can inform cancer treatment. Somatic copy number alterations (CNAs) are important in cancer evolution and are often used as markers, alone or with other somatic mutations, for phylogenetic inferences, particularly in low-coverage DNA sequencing data. Many phylogenetic inference methods using CNAs detected from bulk or single-cell DNA sequencing data have been developed over the years. However, there have been no systematic reviews on these methods. To summarize the state-of-the-art of the field and inform future development, this review presents a comprehensive survey on the major challenges in inference, different types of methods, and applications of these methods. The challenges are discussed from the aspects of input data, models of evolution, and inference algorithms. The different methods are grouped according to the markers used for inference and the types of the reconstructed trees. The applications include using phylogenetic inference to understand intra-tumor heterogeneity, metastasis, treatment resistance, and early cancer development. This review also sheds light on future directions of cancer phylogenetic inference using CNAs, including the improvement of scalability, the utilization of new types of data, and the development of more realistic models of evolution.

## Highlights

• Phylogenetic trees built with copy number alterations are important for understanding the evolution of cancers.
• Reconstructing phylogenetic trees with copy number alterations is challenging.
• A phylogenetic tree can be built using only copy number alterations or by integrating them with other somatic variants.
• Efficient and accurate phylogenetic inference can aid oncological research and offer insights into cancer treatment.

## Graphical abstract

**Figure:**
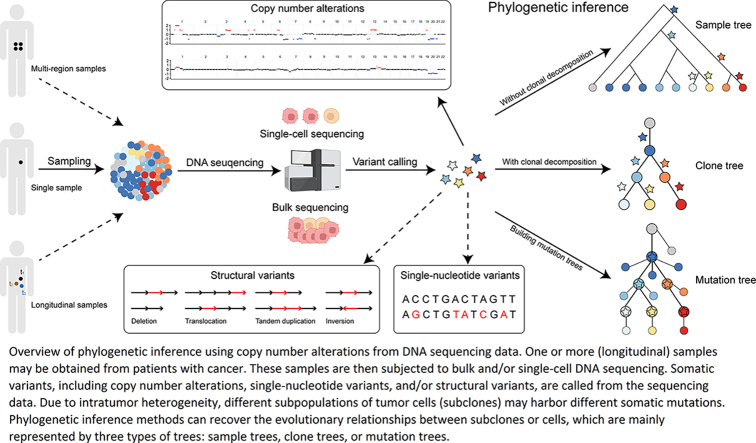

## Introduction

Cancer is a complex genetic disease characterized by the accumulation of diverse somatic mutations and clonal evolution, wherein subpopulations of cells with different mutations evolve under dynamic selection forces.^1^ The presence of distinct cell subpopulations with varying prevalence in the same tumor is called intratumor heterogeneity (ITH), which provides diversity for natural selection and plays a critical role in therapeutic failure.^1^ A better understanding of the evolutionary dynamics of somatic mutations is critical for deciphering ITH and improving the prevention, early diagnosis, and treatment of cancer.^2^^,^^3^ Phylogenetic inference methods, which use computational and statistical techniques to reconstruct lineage trees representing the evolutionary relationships among cells in patient samples, offer a powerful approach to deciphering the complex evolutionary history of somatic mutations in cancer.^4^^,^^5^ Typical uses of cancer phylogenetic trees include determining the order and timing of important evolutionary events in disease progression, such as whole-genome doubling (WGD) and metastatic dissemination,^6^, ^7^, ^8^, ^9^, ^10^, ^11^ revealing the development of therapeutic resistance and potential future progression,^12^^,^^13^ and identifying putative therapeutic vulnerabilities and strategies to circumvent treatment resistance.^14^ Through these diverse applications, phylogenetic inferences can contribute to elucidating cancer mechanisms and genetics involved in tumor initiation, progression, metastasis, and treatment resistance.

Single-nucleotide variants (SNVs) are the most commonly used markers for cancer phylogenetics, followed by copy number alterations (CNAs), which refer to the gain or loss of copies of DNA in large regions of the genome. CNAs occur in many cancers, especially those driven by chromosomal instability, and contribute to ITH.^15^, ^16^, ^17^ The dynamic nature of CNA profiles during cancer progression and treatment underlines the significance of CNAs in understanding cancer cell evolution and treatment efficacy.^18^^,^^19^ Additionally, it is easier to identify CNAs than SNVs because of the larger sizes of copy number changes, which can be determined from low-coverage genome sequencing data commonly acquired using recent single-cell sequencing techniques.^20^^,^^21^ Therefore, it is crucial to build CNA trees, given the biological importance of CNAs in cancer evolution and their suitability for phylogenetic analysis using low-coverage sequencing data.

Previous reviews and comparative studies on cancer phylogenetics have mostly focused on SNV trees.^4^^,^^22^^,^^23^ Although cancer phylogenetic inference methods using CNAs have been briefly summarized in the review on mathematical and computational modeling of cancer evolution,^24^ significant advances have been made in the development of CNA trees in recent years. The latest review on computational approaches for inferring cancer evolution from genomic data at the single-cell level briefly summarized the reconstruction of phylogeny from single cells, including methods for building CNA trees.^25^ However, there have been no comprehensive reviews on cancer phylogenetic inference methods using CNAs detected from bulk or single-cell DNA sequencing data. To summarize the latest advances in this field and guide future development, we conducted a thorough examination of these methods, highlighting the challenges in achieving accurate and efficient inferences. We included methods that integrated CNAs with other markers, such as SNVs and categorized them according to the types of trees inferred. We also discussed the major characteristics of these methods and how they address specific challenges. Finally, we briefly summarized the practical applications of these methods and the limitations of this review.

## Overview of phylogenetic inference methods using copy number alterations

There are two main categories of methods for classical phylogenetic inference: distance- and character-based methods. Distance-based methods often compute pairwise distances between the input data and build a tree from the distance matrix (DM). In contrast, character-based methods use the input data directly to infer the tree, including the maximum parsimony (MP) inference, which aims to find the tree with the minimum number of mutations, and the likelihood-based inference, where the best tree is determined using the maximum likelihood (ML) or Bayesian approach according to the likelihood of trees.^26^ DM methods reduce complex raw data to simple summary statistics and ignore important information in the full dataset.^5^ MP methods are sensitive to convergent or parallel evolution, which can lead to a long-branch attraction artifact.^26^ Likelihood-based inferences, especially the Bayesian approach, can handle more complex evolutionary scenarios and learn complicated lineage-specific rate parameters.^4^^,^^26^

Phylogenetic inferences with CNAs often use the reported copy numbers as input, which can be total (sum of the copies of both homologs), allele-specific, or haplotype-specific (maternal and paternal copies phased into each homolog).^27^, ^28^, ^29^ For a heterogeneous bulk sample, a mixture of distinct clones often leads to fractional copy numbers that can be deconvolved into subclonal integer copy numbers.^30^ Absolute allele-specific integer copy numbers are often called using single-nucleotide polymorphism (SNP) arrays and high-coverage sequencing data, whereas copy numbers detected from shallow whole-genome sequencing (WGS) data are often related to the ploidy of the genome.^20^ CNAs along the genome are frequently represented as vectors of integer copy numbers called copy number profiles (CNPs).

Classical methods developed for species phylogenetics have been directly applied to CNPs to build CNA trees. Commonly used approaches include MP^31^^,^^32^ and DM methods with Euclidean or Manhattan distances.^33^^,^^34^ These methods are easy to implement with available packages. However, they do not use models of evolution to correct hidden changes during distance calculation; hence, they may underestimate the true evolutionary distances.^35^^,^^36^ The variety and unique features of CNA data detected from cancer genomes also inhibit the straightforward use of classical phylogenetic methods in many cases and demand specifically designed approaches.^4^

### Challenges in building trees with copy number alterations

Because phylogenetic inference uses an algorithm to fit data to a model of biological processes,^4^ the major challenges of inference using CNAs can be summarized in terms of data, models, and algorithms. It is also challenging to maintain consistency among data, models, and algorithms, even when solving a specific question on CNA evolution, given the inherent intricacies in many aspects.^4^

#### Issues in copy number alteration detection

CNAs in cancer genomes can be discovered using pre-sequencing and sequencing techniques. Pre-sequencing techniques, such as fluorescence *in situ* hybridization, array comparative genomic hybridization, and SNP arrays, are cheap; however, they are limited to detecting variants in the reference genome used to design probes and have lower resolution.^37^ As high-throughput sequencing data, such as targeted deep sequencing (TDS), whole exome sequencing (WES), and WGS, are becoming more widespread, we will focus on newer methods developed or applicable to CNAs detected from sequencing data. Although CNAs can be detected via ribonucleic acid (RNA) sequencing and assay for transposase-accessible chromatin (ATAC) sequencing,^38^^,^^39^ most phylogenetic inference methods have been developed for DNA sequencing data, which is the focus of this review. Errors in CNA detection often affect downstream phylogenetic reconstruction. For example, an input with excessive false detections generates unreliable phylogenetic inferences. Moreover, the accurate detection of CNAs is challenging for different types of sequencing data and cancer samples.

Sequencing depth directly affects detection accuracy. Low-coverage DNA sequencing data, such as shallow WGS (approximately 0.1× genome coverage), are cost-effective. However, they are error-prone and noisy owing to insufficient read depth, with high missing data rates. Detecting CNAs using shallow WGS faces many challenges, such as repetitive sequences, polymorphisms, variable sample quality, and biases in sequencing procedures.^27^

Patient samples can be either pooled cell populations (bulk) or single cells, which present different challenges to CNA detection. For bulk data comprising a mixture of heterogeneous cancer cells and normal cell contaminants, clonal decomposition is often required to determine the number of clones and clone proportions (the fraction of cells in each sample belonging to each clone) from aggregated signals.^4^^,^^24^ Most CNA detection methods for bulk data generate simplifying assumptions to avoid the complexities of clonal decomposition. Additionally, methods that infer clone-specific copy numbers may miss rare CNAs, especially in datasets with low coverage and/or high normal cell proportions.^30^^,^^40^^,^^41^ Although single-cell sequencing circumvents the need to infer clone structure, it is more expensive, and the data generated is still very noisy.^33^^,^^42^ Because there is only a limited quantity of DNA in a cell, there may be sequencing artifacts, allelic dropouts, cycling cells, and DNA amplification errors from different whole-genome amplification procedures.^43^^,^^44^ These instances make it challenging to detect CNAs accurately.^21^^,^^29^

Different tumor sampling approaches also pose challenges in CNA detection. Most bulk tumor samples are solid biopsies that were either fresh (frozen) or archival formalin-fixed and paraffin-embedded (FFPE). FFPE samples are commonly available for diagnostics, but they frequently lack matched reference samples, have low DNA quality owing to degradation, and are often characterized using shallow WGS.^20^^,^^27^ Many solid tumor samples are single biopsies obtained at a given time point, which are weakly informative for detecting CNAs from minor clones and reconstructing the subclonal architecture.^30^^,^^45^ With decreasing sequencing costs, multi-regional and temporal sampling, which can better characterize CNAs and ITH, are becoming more common, such as those previously reported in samples obtained from the human tumor atlas network^46^ and long-term surveillance of inflammatory bowel disease and Barrett's esophagus.^32^^,^^47^ Methods for joint copy number decomposition from multiple samples have also demonstrated good performance^40^^,^^41^; however, they still have restrictive assumptions that limit more general applications.

At the same time, noninvasive liquid biopsy techniques are being developed rapidly and are entering clinical settings.^48^ Liquid biopsies allow easier sampling and isolation of circulating tumor DNA (ctDNA) for the serial monitoring of cancer prognosis and diagnosis, minimal residual disease, and recurrent or metastatic disease.^49^ Although studies have suggested the feasibility of utilizing CNAs to track metastasis and treatment responses, detecting CNAs in ctDNA samples with a low tumor fraction remains challenging.^19^^,^^50^

#### Difficulties in modeling copy number alterations

The model of evolution plays an important role as the basis for phylogenetic inference; however, it is challenging to propose a model of CNAs that maintains a good trade-off between biological realism and complexity.^21^

The simplest model commonly used in cancer phylogenetics is the infinite sites model (ISM), which assumes that each site has only two states and each site changes state at most once.^22^^,^^51^ Specifically, the site evolves without homoplasy, and tumor evolution is consistent with a perfect phylogeny, where all sites in the input are compatible with the tree. ISM implies that each node in the phylogenetic tree inherits all mutations in its ancestors, and each mutation occurs only once in a subtree. ISM has been frequently used to infer phylogeny from SNVs but is often violated for CNAs, which usually have more than two states.^21^

CNAs can also vary from small focal duplications/deletions to large arm- or chromosome-level gains/losses and WGD, which are likely to cause overlaps, back mutations, and complex dependencies.^15^^,^^17^^,^^52^ CNA generation may involve complex mutational events, such as chromothripsis, breakage-fusion-bridges, and template insertions.^53^ CNAs may undergo convergent or parallel evolution, which generates similar patterns that obscure the true evolutionary relationships.^54^ Punctuated evolution is also common for CNAs, where many genome rearrangement events occur in a very short time, causing a rapid accumulation of alterations.^34^

To address these complexities, some methods transform copy numbers into the presence or absence of changes (breakpoints) to simplify spatial correlations across sites.^35^^,^^55^ Because breakpoints are tp53 to overlap despite the potential presence of multiple CNAs at the same site, the ISM is well-approximated. However, it does not use full copy number data and may lead to information loss. Because CNAs have two endpoints, this simplification will also cause artificial duplication, create pairwise dependencies, and mislead phylogenetic inferences.^55^

The Dollo model is an extension of the ISM. It is often used for SNV evolution, which assumes that a mutation may be gained at most once but lost multiple times in different lineages. To constrain SNV losses to loci with a decreased copy number due to CNAs, a loss-supported Dollo model was proposed, but it remains an open problem how to extend into a unified model for SNVs and CNAs.^56^

To allow for more copy number changes, the infinite alleles model (IAM) has been proposed, which treats a site as a character whose state corresponds to the possible number of copies at that site, where an allele at a given site corresponds to a particular copy.^51^ It assumes that a character may change state more than once on the tree and change to the same state at most once, which leads to non-homoplasy and a multi-state perfect phylogeny.^51^ However, IAM violations are likely to occur in highly rearranged cancer genomes with extensive CNAs.^21^^,^^51^

A few methods use the finite-sites model (FSM), which allows a site to change states multiple times.^21^^,^^57^, ^58^, ^59^ Continuous-time Markov chain is a common FSM, which corrects multiple hits at the same site and allows gain/loss to occur more than once at any site.^21^^,^^26^^,^^57^ The Markov chain assumes that sites across the genome are independently and identically distributed. This allows efficient computational inferences, such as the likelihood computation required by the ML and Bayesian method, by taking the product across all sites. However, analyses at individual sites are more likely to be affected by errors in detecting CNAs, and analyses on a larger scale may become intractable.^21^

Other methods use copy number transformation (CNT) models that allow the computation of the minimum event distance (MED) between two CNPs, which corresponds to the shortest sequence of events that transforms one CNP into another.^60^^,^^61^ The CNT model can handle horizontal dependencies caused by multiple overlapping CNAs. Hence, it is less likely to be affected by convergent or parallel evolution. It has also been extended to allow weights on CNAs of different positions, sizes, and types.^36^^,^^62^, ^63^, ^64^ However, these models only consider individual genomic units (such as genes, chromosomes, or genomes) or genomic alterations contiguous with the reference genome, which do not explicitly represent translocations, inversions, or complex genomic rearrangements.

Some models for tandemly repeated DNAs (e.g., microsatellites) and multigene families also seem relevant to CNAs, such as the stepwise mutation model of microsatellites and the birth-death model of multigene families.^21^^,^^65^ The stepwise mutation model assumes that a mutation can alter its copy number to one of the ordered discrete microsatellite states. The birth-death model of multigene families assumes that a gene undergoes duplication (birth) or loss (death), leading to the expansion and contraction of family sizes across different species. They may be suitable for modeling CNAs; however, it is still unclear how to adapt these models.

#### Difficulty of tree inference problems

Many phylogenetic problems are intractable and lack efficient polynomial-time solutions. The copy-number tree problem, which involves the construction of a phylogenetic tree whose leaves are labeled by the given CNPs and the assignment of CNPs to the internal nodes under an MP criterion so that the sum of distances over all edges is minimum, was proven to be nondeterministic polynomial time (NP) hard, which belongs to the most challenging categories of computational problems.^66^ The parsimonious clone tree integration (PACTION) problem, which requires inference of a set of clones and a combined clone tree, given the clone trees and clonal proportions of SNVs and CNAs, so that the combined clone tree is the refinement of the SNV tree and CNA tree with minimal errors, has also been proven to be NP-hard.^67^ The copy-number tree mixture deconvolution problem, which requires deconvolution of the fractional copy numbers of mixed tumor samples into clones with integer copy numbers and the inference of a phylogenetic tree of these clones with the fewest number of CNAs, is likely to be NP-hard, although it remains open.^68^ The rectilinear Steiner minimum tree problem, which involves identifying a minimum weight tree that includes all observed data and unobserved Steiner nodes with a rectilinear metric, is NP-complete and more challenging than NP-hard problems.^69^ The Steiner problem in phylogeny, which involves finding a Steiner minimal tree with the minimum possible length over all trees that contain the observed data as leaves, is NP-complete.^70^ The multi-state perfect phylogeny mixture deconvolution problem, which requires the inference of a perfect phylogeny with each edge labeled with exactly one character-state pair and no pair appearing more than once in the phylogeny, as well as the clonal proportions for each sample, given the SNVs and allele-specific subclonal copy numbers along with their frequencies, is NP-complete, even for two samples and two sites.^51^

The difficulty of these problems makes it challenging to obtain global optima and scale the algorithms to high numbers of CNAs and samples. As the sample space for the phylogenetic tree grows super-exponentially with an increasing number of leaves, the large number of CNPs from thousands of single cells significantly increases the computational burden. Because of model complexity, methods that jointly consider CNAs and SNVs generally do not scale to large numbers of mutations or samples in large multi-sample sequencing studies.^71^ The ML and Bayesian methods require computing the likelihood, which incurs huge computational costs for large datasets.^26^ For example, standard Markov chain Monte Carlo (MCMC) sampling for Bayesian inference enables the exploration of all possible trees and related parameters, accompanied by inherent uncertainty measures; however, the number of steps required to obtain accurate results typically increases exponentially with the number of leaves in the tree.

### Classification of tree-building methods using copy number alterations

**Figure 1:**
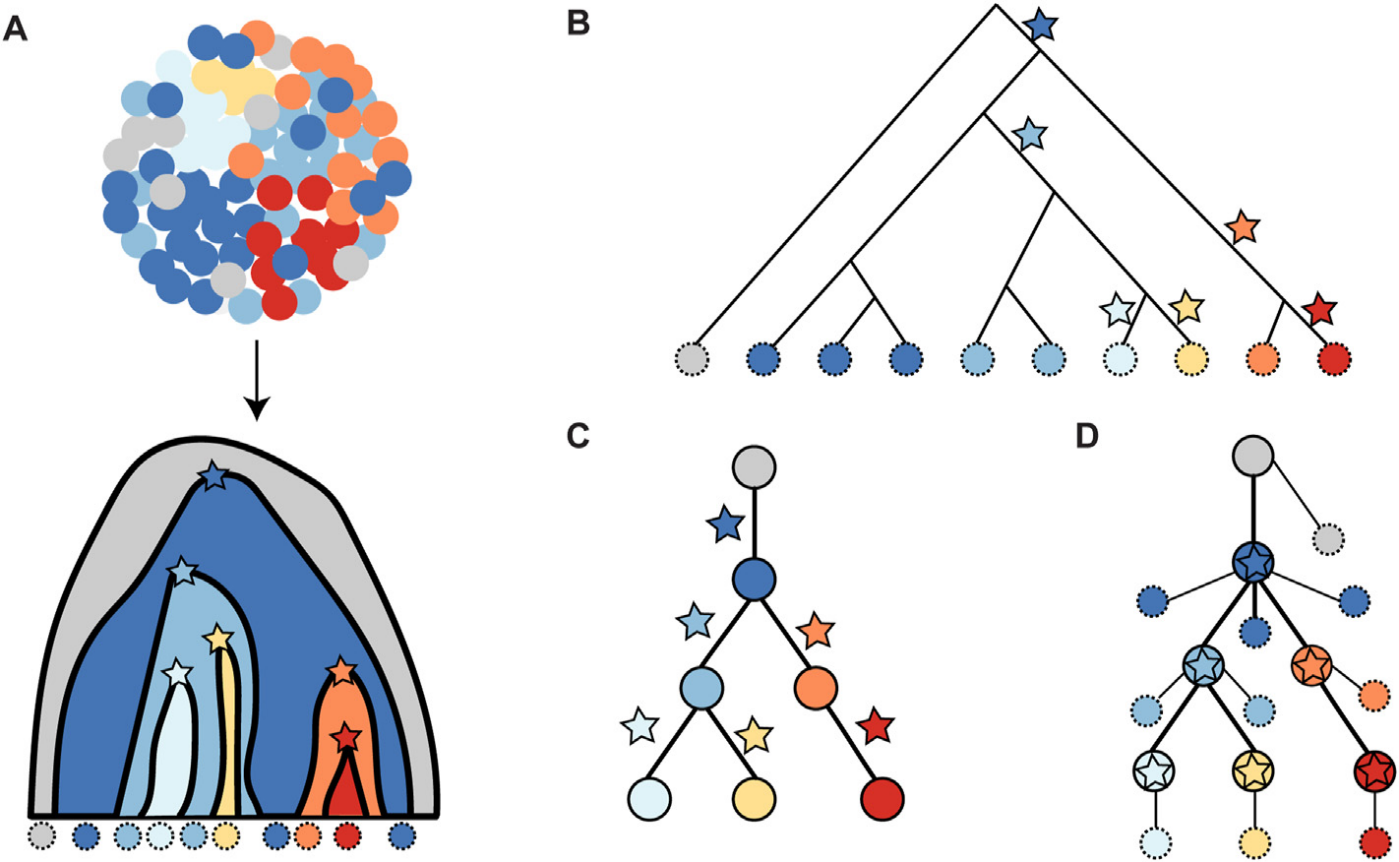
Major representations of copy number trees. (A) True phylogeny of 10 cells (dashed circles) sampled from seven clones of a heterogeneous tumor, where additional mutations (copy number alterations) for each clone (distinguished by a different color) are represented by a star with the same color as the clone. The initial clone with normal cells is depicted in light gray. (B) The sample tree represents the relationships among the sampled cells. (C) The clone tree, with the branches annotated by the mutations, leads to each clone (solid circle with a star). (D) The mutation tree, where each node is represented by the mutational events defining each clone. The sampled cells are attached as leaves.

Many methods have been developed to address the challenges of phylogenetic inference using CNAs for data obtained from different platforms. Most methods have been designed for bulk data, whereas an increasing number of methods have been developed for single-cell data in recent years. These methods represent inferred trees in various formats. The cancer phylogenetic tree is naturally rooted in a normal diploid cell or clone with no mutations and runs down the tree from the root. Branch lengths may represent the number of mutations (CNAs), time, or may be arbitrary.^72^ Depending on the meaning of the non-root nodes, the trees inferred using different methods have three major formats: sample trees, clone trees, and mutation trees [Figure 1].

In a sample tree, the leaves represent all observed single cells or bulk samples. When built for bulk data, the bulk sample is assumed to be homogeneous with only one clone, which holds true when the detected CNAs represent the dominant clone in a sample. As this assumption is likely to be violated, it is more appropriate to perform clonal decomposition and build clone trees when possible.^73^ The clone tree illustrates the relationship between clones of cell populations, distinct sets of mutations from each clone, and clone proportions, which are mostly non-binary unless explicitly stated. As the order and evolutionary history of mutational events may appear more relevant than fully resolved cell genealogies, the mutation tree has been used for single-cell data, where each internal node represents a set of mutational events, and cells are attached as leaves.^22^ In addition to inferring the order of CNA events, the mutation tree helps determine mutually exclusive or co-occurring CNAs in the same clone.^22^^,^^57^^,^^74^

Some methods build trees using CNAs alone, whereas others attempt to integrate other types of data, such as SNVs and structural variants (SVs). CNAs are closely related to SVs through chromosomal instability, which often causes structural or numerical chromosomal alterations in cancer genomes.^75^ Owing to their extreme complexities, SVs have been incorporated into phylogenetic inferences only recently.^76^, ^77^, ^78^ Herein, we divided the methods into three types according to the markers used: those based only on CNAs, those integrating CNAs and SNVs, and those integrating CNAs and SVs. For each type, some methods build sample trees and do not perform subclonal reconstruction, whereas others infer clones or mutation trees. In the following sections, we briefly discuss each type of method in terms of the data, model, and algorithm used.

## Phylogenetic inference methods based solely on copy number alterations

Most methods based solely on CNAs do not perform clonal decomposition to address ITH from bulk data because it is assumed that the input CNAs have already been decomposed, or it is not feasible to obtain an accurate clonal architecture from the low-coverage input data [Table 1].

**Table 1:** Software tools for cancer phylogenetics using CNAs alone.

Tool	Data	Model	Algorithm	Pros	Cons	Availability
**Methods without clonal decomposition**						
MEDICC2	Allele-/haplotype-specific/total CN	CNT	DM	Scalable to thousands of cells, inferring WGD	Requires robust data pre-analysis	https://bitbucket.org/schwarzlab/medicc2
CNT-ILP	Total CN	CNT	MP	Applicable to shallow WGS data	Not tested on real data	https://github.com/raphael-group/CNT-ILP
Lazac	Total CN	CNT	MP	Scalable to thousands of cells	Limited tests on real data	https://github.com/raphael-group/lazac-copy-number
*MEDALT*	Total CN	CNT	MP	Scalable to thousands of cells	Does not generate a proper phylogenetic tree	https://github.com/KChen-lab/MEDALT
CNETML	Total/haplotype-specific CN	FSM	ML	Applicable to shallow WGS data and longitudinal samples	Not designed for WES data	https://github.com/ucl-cssb/cneta
*NestedBD*	Total CN	FSM	Bayesian	Infers branch lengths from single-cell data	Only applicable to a small number of cells	https://github.com/Androstane/NestedBD
*Sitka*	Total CN	ISM	Bayesian	Scalable to thousands of cells	Information loss from data transformation	https://github.com/UBC-Stat-ML/sitkatree
**Methods with clonal decomposition**						
CNT-MD	Fractional total CN	CNT	MP	Deciphers CNA-ITH	Potential overfitting of the number of clones	https://github.com/raphael-group/CNT-MD
SCS_ deconvolution	Fractional total CN	No	MP	Combines bulk and single-cell data	Lack of real data	https://github.com/CMUSchwartzLab/SCS_deconvolution
DEVOLUTION	Mutated clone fraction	Jukes-Cantor	ML, MP	A standardized inference framework for bulk data	Requires robust data pre-analysis	https://github.com/NatalieKAndersson/DEVOLUTION
**Methods for building mutation trees**						
*SCICoNE*	Binned read count	FSM	Bayesian	Joint tree inference and CN calling	Requires robust data pre-analysis	https://github.com/cbg-ethz/SCICoNE
*CONET*	Binned read count	FSM	Bayesian	Joint tree inference and CN calling with enhanced efficiency	Requires robust data pre-analysis	https://github.com/szczurek-lab/CONET

The methods developed for single-cell data are shown in italics. CN: Copy number; CNA: Copy number alteration; CNETML: Copy number evolutionary tree maximum likelihood; CNT: Copy number transformation; CNT-ILP: Copy-number tree-integer linear programming; CNT-MD: Copy-number tree-mixture deconvolution; CONET: Copy number event tree; DM: Distance matrix; FFPE: Formalin-fixed and paraffin-embedded; FSM: Finite sites model; ISM: Infinite site model; ITH: Intratumor heterogeneity; Lazac: Large-scale analysis of zero agnostic copy number; MEDALT: Minimal event distance aneuploidy lineage tree; MEDICC2: Minimum event distance for intra-tumour copy-number comparisons 2; ML: Maximum likelihood; MP: Maximum parsimony; WGD: Whole-genome doubling; WGS: Whole-genome sequencing.

### Methods without clonal decomposition

Methods based solely on CNAs without clonal decomposition assume that the input copy numbers are clonal, and hence, are integers.

MEDICC (Minimum Event Distance for Intra-tumour Copy-number Comparisons)^61^ and its improved version, MEDICC2,^36^ reconstruct CNA trees under minimum evolution criteria from allele-specific CNPs detected from either bulk or single-cell data. They can determine the optimal phasing of major and minor alleles. MEDICC2 can also deal with haplotype-specific CNPs pre-phased using other methods and total CNPs. MEDICC and MEDICC2 not only infer the tree topology using DM methods based on pairwise MEDs, but also infer ancestral CNPs and branch lengths by assuming that the total number of events along the tree is minimal. They can also compute summary statistics from the inferred trees to numerically quantify tumor heterogeneity. MEDICC first proposed a CNT model that assumed a copy number duplication/deletion event that may affect contiguous segments of variable sizes. MEDICC2 incorporates WGD and loss-of-heterozygosity into the model and implicitly accounts for arm- or chromosome-level gain/loss by grouping segments on the same arm or chromosome, allowing it to infer WGD events. With a fast algorithm to minimize the MED between two CNPs and their ancestors and an efficient parallelization strategy to split pairwise distance computations into smaller parallelizable chunks, MEDICC2 can efficiently scale to thousands of cells.

CNT-ILP (Copy-Number Tree-Integer Linear Programming)^66^ infers a binary CNA tree from the total CNPs by solving the NP-hard copy-number tree problem using ILP. Each edge in the tree is labeled by the sequence of events that change the CNP at the parent node to that at the child node and is associated with a cost. It adopts the CNT model proposed in MEDICC^61^ and only applies to segmental duplications and deletions. CNT-ILP was a proof-of-principle study and hence only tested on simulated data of up to six samples and 40 sites. Lazac (Large-scale analysis of zero agnostic copy number) extended the CNT model to allow the amplification or deletion of zero-copy number regions, which is not biologically realistic yet makes distance a metric to achieve more efficient computation.^79^ Lazac built an MP tree based on a linear-time approximation algorithm on a zero-agnostic CNT model that outperformed several other methods in terms of accuracy and speed on simulated and real single-cell datasets.

MEDALT (Minimal Event Distance Aneuploidy Lineage Tree)^80^ infers a rooted directed minimal spanning tree (MST) that optimally connects sampled single cells with the fewest CNAs from the total CNPs using a classical combinatorial algorithm. The tree has no unobserved internal ancestral nodes. Each node represents a cell, and each edge signifies the kinship between two cells, the younger of which is indicated by an arrow. It also uses the CNT model of duplications and deletions^61^ but computes the MED with a linear-time greedy algorithm similar to that used previously.^81^ It uses statistical lineage speciation analysis to facilitate the discovery of fitness-associated CNAs and genes from trees and to identify potential CNAs with parallel or convergent evolution. Owing to the existence of a polynomial runtime algorithm for inferring an MST rather than a Steiner tree, MEDALT can scale to thousands of cells.

CNETML (Copy Number Evolutionary Tree Maximum Likelihood)^59^ is the first method that can jointly infer the tree topology, node ages, and mutation rates of longitudinal patient samples from total CNPs. Using a Markov model of duplication and deletion, CNETML infers a tree from the total or haplotype-specific CNPs of multiple samples using classical ML inference approaches. It utilized temporal information from longitudinal samples to estimate the timing and rates of CNAs, which are important parameters in carcinogenesis, but have rarely been studied. It can also build trees based on relative total copy numbers, which helps alleviate the effects of WGD. The input CNPs are assumed to be called from shallow WGS data and hence cover the whole genome. Although this can be applied to CNAs detected from WES data, acquisition bias may need to be addressed to obtain more reliable results.

Both NestedBD^58^ and Sitka^55^ infer a cell tree from the total CNPs detected in single-cell data using the Bayesian method. NestedBD can infer topologies (binary), ancestral CNPs, and branch lengths, which represent the relative time of evolution, by assuming a random local clock to allow varying rates among branches.^82^ Sitka, on the other hand, can infer a multifurcating tree without branch length and represent each internal node as an acquisition of a single breakpoint or copy number change. NestedBD uses a birth-death model of duplication and deletion, a special type of Markov chain where transitions from a state *i* can only go to the adjacent states *i* + 1 or *i* − 1, where a birth (death) event corresponds to a duplication (deletion).^58^ Sitka uses a lossy binary encoding of CNAs that transfers raw copy numbers to the presence/absence of breakpoints to simplify site dependencies, enabling scalable MCMC sampling under ISM. Sitka also introduced a matrix to model errors in copy number calls at the locus-specific and global levels with different false-positive and false-negative rate parameters. Moreover, Sitka can call SNVs and place them on an inferred CNA tree by incorporating copy number information to overcome the difficulty of calling SNVs from low-coverage single-cell WGS data. However, Sitka infers CNAs and SNVs from the same set of cells, which leads to SNV calls with high missing rates.

### Methods with clonal decomposition

Only a few methods perform clonal decomposition of CNAs together with phylogenetic inference because of the difficulty in obtaining clone-specific copy numbers.^30^^,^^40^^,^^41^

CNT-MD (Copy-Number Tree-Mixture Deconvolution)^68^ reconstructs a binary clone tree under the MP criterion from bulk sequencing data of multiple tumor samples by solving the copy-number tree mixture deconvolution problem. The edges in the tree are labeled as interval events that correspond to duplications or deletions of an interval in the CNP or the cost of these events. It uses the CNT model in MEDICC^61^ and minimizes the distance between the observed and inferred fractional copy numbers using a heuristic coordinate descent algorithm with alternating integer linear programming and linear programming steps. CNT-MD requires a prespecified number of clones *n* and must run with varying values of *n* in practice, which may overfit the data with the current simple approach of selecting *n*.

SCS_deconvolution^83^ reconstructs a multifurcating cell tree under the minimum evolution criteria using both bulk and single-cell data. It uses a mixed-membership model to formalize CNA deconvolution as a nonnegative matrix factorization problem, extends the problem to minimize the phylogenetic distance between single cells and inferred clones, and incorporates a minimum evolution tree cost in the problem objective. Similar to CNT-MD,^68^ it uses a coordinate descent algorithm with mixed ILP to solve the optimization problem. SCS_deconvolution demonstrates the feasibility of CNA deconvolution and phylogenetic inference by combining limited amounts of bulk and single-cell data. Since most single-cell studies to date have sampled many cells from a few patients rather than from large cohorts, it is difficult to find real applications of SCS_deconvolution because of the lack of applicable datasets.

DEVOLUTION^84^ infers a clone tree from CNAs (and SNVs, if available) detected from multi-regional bulk genotyping data, including SNP arrays, TDS, WES, and WGS. Given an input matrix of the mutated clone fraction that represents the proportion of cells carrying each alteration in each sample, DEVOLUTION performs subclonal decomposition based on the clustering results of the alterations to obtain an event matrix upon which phylogenetic reconstruction is based. The event matrix, which represents the distribution of alterations among the involved subclones, can be improved using user-specified rules for illicit alterations. The tree is then reconstructed from the event matrix using classical ML (with the Jukes-Cantor model^85^) and MP methods, which allow the inference of ancestral states and branch lengths that represent the number of alterations. Although DEVOLUTION can handle missing data in the input, it requires a robust pre-analysis of the input data. Moreover, although it identifies all unique alterations across samples, it cannot determine the order of events overlapping with each other across samples.

### Methods for building mutation trees

Both SCICoNE^74^ and CONET (Copy Number Event Tree)^57^ were developed for single-cell DNA sequencing data. Each method jointly reconstructs a mutation tree of CNAs, detects breakpoints, and calls CNPs. With joint inference, they are more robust against noise in copy number detection from low-coverage data. In the multifurcating mutation tree, each internal node was labeled with either a vector of CNA events by SCICoNE or pairs of breakpoints by CONET. Given the binned read counts, they both employ a probabilistic model and MCMC sampling for inference. Both CNA models are FSMs that allow repeated amplifications/deletions to account for the overlap and re-occurrence of CNAs. SCICoNE detects breakpoints by aggregating evidence across all cells and segments the genome into regions by merging bins between consecutive breakpoints, which reduces the number of sites and increases the efficiency of inference. CONET identifies candidate breakpoints for each cell by calculating the differences between corrected per-bin read counts and integrating breakpoints into the model to avoid modeling exact copy number changes. This reduces the search space for possible models and enhances the inference efficiency of CONET together with an efficient MCMC implementation. CONET requires normalized data with a given basal ploidy, whereas SCICoNE uses model comparisons to select the best ploidy for the given data. Both methods require data pre-processed with GC-content and mappability corrections to address the substantial noise present in single-cell data. Therefore, residual confounding effects of pre-processing can directly affect downstream analyses and complicate inferences.

## Phylogenetic inference methods integrating copy number alterations with single-nucleotide variants

CNAs and SNVs are closely related to each other during cancer progression, as CNAs may overlap with SNVs, resulting in SNV gains or losses. Hence, relying solely on a single type of marker for phylogenetic inference may overlook the intricacies in the interplay between CNAs and SNVs. Integrating SNVs and CNAs aids the reconstruction of phylogenies that better capture the evolutionary history of cancer genomes. Although some analyses have manually annotated CNAs on an SNV tree,^6^^,^^86^ the joint modeling of SNVs and CNAs in phylogenetic inference is common with bulk sequencing data and has been reviewed previously.^4^^,^^22^^,^^45^ These methods often use SNVs as major markers and integrate CNAs with SNVs to obtain more accurate variant allele frequencies (VAFs) for joint clonal decomposition and tree-building.

Only a few methods have been developed for single-cell sequencing, primarily because of the challenges of simultaneously detecting CNAs and SNVs in the same set of single cells [Table 2]. SNVs and CNAs differ in mechanism and scale, with SNVs involving single base pairs and CNAs encompassing regions of diverse sizes. Therefore, they are often detected using distinct technologies. Different features of whole-genome amplification techniques in single-cell DNA sequencing allow for the reliable measurement of either SNVs or CNAs.^87^ Some targeted technologies detect sequence-specific genomic regions, which typically comprise cancer-related genes, at a high depth (approximately 50× for hundreds of amplicons from thousands of cells), allowing the accurate identification of SNVs, but not CNAs.^6^ Targeted sequencing data from the Mission Bio Tapestri platform allows the detection of SNVs and CNAs from the same cells yet has limited coverage of the genome.^88^

**Table 2:** Software tools available for cancer phylogenetics using CNAs and SNVs.

Tool	Data	Model	Algorithm	Pros	Cons	Availability
**Methods without clonal decomposition**						
*SCARLET*	Read count, CNA tree	Dollo	ML	First method applicable to hundreds of cells	Requires CNAs and SNVs from the same set of cells	https://github.com/raphael-group/scarlet
**Methods with clonal decomposition**						
PhyloWGS	Read count, clonal allele-specific CN	ISM	Bayesian	First automated method applicable to bulk WGS data of single or multiple samples	Requires subclonal CNAs as input	https://github.com/morrislab/phylowgs
Canopy	VAF, fractional allele-specific CN	ISM	Bayesian	Applicable to bulk data from multiple samples	Requires robust data pre-analysis	https://github.com/yuchaojiang/Canopy
PhylogicNDT	Purity, read count, allele-specific CN	ISM	Bayesian	Applicable to bulk data from single or multiple samples	Requires robust data pre-analysis	https://github.com/broadinstitute/PhylogicNDT
Pairtree	Read count	ISM	Bayesian	Scalable to as many as 30 subclones	Mainly tested on WES data	https://github.com/morrislab/pairtree
CONIPHER	Read count, fractional allele-specific CN	ISM	MP	Incorporates error correction	Mainly tested on WES data	https://github.com/McGranahanLab/CONIPHER
SPRUCE	VAF, clonal allele-specific CN	IAM	MP	Applicable to bulk data from multiple samples	Requires subclonal CNAs as input	https://github.com/raphael-group/spruce
SIFA	Read count	ISM	Bayesian	Integrates SNVs, CNAs, and phylogeny within a single framework for bulk data	Designed for WGS data only	https://github.com/zengliX/SIFA
*BiTSC*^*2*^	Read count	ISM	Bayesian	Integrates SNVs, CNAs, and phylogeny within a single framework for single-cell data	Requires CNAs and SNVs from the same set of cells	https://github.com/ucasdp/BiTSC2
*SCsnvcna*	SNV genotype, CNA tree	Dollo	Bayesian	Applicable to SNVs and CNAs from independent single-cell data sets	Requires robust data pre-analysis	https://github.com/compbio-mallory/SCsnvcna
PACTION	SNV tree, CNA tree	No	MP	Integrates available SNV trees and CNA trees	Requires robust data pre-analysis	https://github.com/elkebir-group/paction
**Methods for building mutation trees**						
*COMPASS*	Read count	Dollo	MAP	Scalable to thousands of cells	Designed for target sequencing data only	https://github.com/cbg-ethz/COMPASS

The methods developed for single-cell data are shown in italics. BiTSC^2^: Bayesian inference of tumor clone tree by joint analysis of single-cell single-nucleotide variant and copy number alteration; CN: Copy number; CNA: Copy number alteration; COMPASS: Copy number and mutations phylogeny from amplicon single-cell sequencing; CONIPHER: Correcting noise in phylogenetic evaluation and reconstruction; IAM: Infinite allele model; ISM: Infinite site model; MAP: Maximum a posteriori probability; ML: Maximum likelihood; MP: Maximum parsimony; PACTION: Parsimonious clone tree integration; PhylogicNDT: Phylogic N-dimensional with timing; SCARLET: Single-cell algorithm for reconstructing loss-supported evolution of tumors; SNV: Single-nucleotide variant; SIFA: Subclone identification by feature allocation; SPRUCE: Somatic phylogeny reconstruction using combinatorial enumeration: VAF: Variant allele frequency; WES: Whole exome sequencing; WGS: Whole-genome sequencing.

### Methods without clonal decomposition

SCARLET (Single-Cell Algorithm for Reconstructing Loss-supported Evolution of Tumors)^56^ is the first method to integrate SNVs with CNA-based tumor phylogeny for single-cell DNA sequencing data. It proposes a loss-supported Dollo model to support the SNV loss caused by CNAs, which depends on the observed and ancestral CNPs that cannot be directly measured. Thus, a CNA tree must be obtained separately from existing methods, from which the supported loss sets of SNVs for pairs of CNPs can be derived. It also takes the observed total and variant read counts as input while accounting for the typically high rates of errors and missing data in single-cell sequencing. Moreover, SCARLET refines the CNA tree by resolving multifurcations with an ML approach to maximize the likelihood of the variant read counts, given the total counts, which are solved using integer linear programming. However, because SCARLET has only been tested on high-coverage data with hundreds of cells,^6^ it remains unclear how it could scale to thousands of cells. Furthermore, SCARLET requires SNVs and CNAs to be from the same set of cells, which is uncommon in datasets obtained from current technologies.^87^

### Methods with clonal decomposition

Most subclonal reconstruction methods correct the VAFs of SNVs for tumor purity (the fraction of cancer cells in the sample) and CNAs to infer the cancer cell fractions (CCFs) of these SNVs, which represent the proportions of cells among all cancer cells carrying specific SNVs.^45^ Some methods also calculate the mutant cell frequency or cellular prevalence, which is the proportion of cells in a sample that carry a specific mutation, including both normal and tumor cells. Assuming that mutations with comparable frequencies are present in a similar set of cells resulting from clonal expansion at a similar time, mutations are clustered into different subclonal clusters.^71^^,^^89^ Clone trees can then be constructed by ordering and nesting subclonal cluster CCFs based on evolutionary principles for constraining lineage relationships, such as the pigeonhole principle, which states that the sum of subpopulations cannot exceed the CCF or mutated clone fraction of their ancestors.^24^^,^^71^^,^^90^ Most clustering-based methods rely on the ISM and the weak parsimony assumption, which implies that most SNVs with detectable VAFs are associated with a few subclonal lineages.^45^^,^^72^^,^^91^

PhyloWGS^91^ is the first automated method that can perform subclonal reconstruction using both CNAs and SNVs by converting sites affected by CNAs into pseudo-SNV sites. It is an extension of Phylosub,^92^ a non-parametric Bayesian MCMC method that infers clone trees and only works on copy-neutral genomic regions. PhyloWGS is applicable to regions with CNAs by incorporating subclonal allele-specific copy numbers and frequencies that must be obtained from existing CNA subclonal reconstruction methods.

Canopy^72^ is another Bayesian MCMC method used to infer clonal phylogeny by considering both SNVs and allele-specific CNAs across all samples. It infers a bifurcating clone tree, where SNVs and CNAs emerge along the branches and form subclones that are represented as leaf nodes, with the non-bifurcating and mutation-free leftmost branch being assumed to represent normal cells. Canopy extends the ISM to CNAs by assuming that CNAs with the same breakpoints and the same copy numbers across all samples resulted from a single CNA event that occurred exactly once. Canopy permits subclonal CNAs and treats overlapping and nested CNAs with different breakpoints or copy numbers as separate evolutionary events. Moreover, it determines the temporal ordering and phase of SNVs within CNAs. It also employs a preclustering initialization step for SNVs to improve their robustness to noise and significantly decrease the computation time.

PhylogicNDT (Phylogic N-dimensional with timing)^93^ includes a suite of tools for inferring clones, phylogenies, timing of somatic events, and growth dynamics of tumor cell populations. First, clustering is applied to estimate the number of clones and posterior clonal frequencies from absolute CNPs, joint SNV calls, and tumor purity values. An MCMC Gibbs sampling method is then used to infer the ensemble of the most likely trees. PhylogicNDT can integrate WGS and WES data from single or multiple samples from the same patient at both the individual and cohort levels.

As most clone tree reconstruction methods become highly inaccurate on datasets with many subclones or cancer samples, Pairtree^89^ adopts a more efficient Bayesian approach to infer clone trees that can contain as many as 30 subclones based on sequencing data from up to 100 samples. It models variant and reference read counts for each SNV using a binomial sequencing noise model with CNA correction. Pairtree also estimates the subclonal frequency of a mutation, which is the proportion of cells in each sample carrying a mutation, by correcting the mutation's VAF for overlapping CNAs. Using the Pairs Tensor to capture pairwise mutation relationships, Pairtree can search the tree space more efficiently using MCMC and detect mutations that violate the ISM or technical issues corrupting the observed data.

Like Pairtree, CONIPHER (COrrecting Noise in PHylogenetic Evaluation and Reconstruction)^71^ can also scale tumors with many samples and clusters in a time frame on the order of minutes using bulk sequencing data. CONIPHER assumes that SNVs and CNAs are available from existing detection methods and that all SNVs must have read counts provided for each sample. CONIPHER accounts for data noise/uncertainty and provides an efficient pipeline in four major steps: (1) cluster nesting to form an ancestral graph, (2) tree growth by pruning the ancestral graph to obtain a spanning tree, (3) enumerating alternative trees in the solution space, and (4) computing the subclone proportions. It improves mutation assignment by removing biologically improbable clusters driven either by likely erroneous SNVs or subclonal CNAs and correcting for SNV losses caused by CNAs. CONIPHER extends the CCF to phylogenetic CCF, representing the fraction of cancer cells either carrying a mutation or whose ancestors carry the mutation before mutation loss. The tree reconstruction stage computes the tree(s) that generate the minimum nesting error or consist of branches shared most frequently across alternative trees, which can also take the CCFs inferred from other mutation clustering methods as the input.

Rather than assuming the ISM, SPRUCE (Somatic Phylogeny Reconstruction Using Combinatorial Enumeration)^51^ formulates and solves the multi-state perfect phylogeny mixture deconvolution problem under the IAM. It requires subclonal allele-specific copy numbers and frequencies, similar to PhyloWGS,^91^ and infers constrained spanning trees using combinatorial enumeration. As the solution space increases rapidly with the number of characters or sites, it is only applicable to a small number of samples and mutations.

Both SIFA (subclone identification by feature allocation)^94^ and BiTSC^2^ (Bayesian inference of Tumor clone Tree by joint analysis of Single-Cell SNV and CNA)^95^ apply a Bayesian MCMC approach to simultaneously estimate the subclonal genotype matrices of CNAs and SNVs aside from the clone tree and clonal proportions from the observed total and variant read counts. SIFA uses a unified Bayesian feature allocation model for bulk sequencing data under several assumptions regarding the evolutionary process of cancer, such as ISM, sample independence, and each CNA occurring at most once in a particular subclone and being passed down to descendant subclones. SIFA applies only to WGS data because it assumes a Poisson distribution when modeling the total number of reads at a locus. BiTSC^2^ generalizes the SIFA model for single-cell data by accounting for the overlap of CNAs and SNVs, modeling allelic dropouts, missing data, and sequencing errors common in single-cell DNA sequencing. BiTSC^2^ is the first method to fully integrate SNVs and CNAs during phylogenetic references from single-cell DNA sequencing data. It robustly performs when dealing with low-coverage data and varying missing rates, with efficacy demonstrated on datasets containing at most 500 cells and coverage as low as 3×. However, BiTSC^2^ assumes that genomic coverage is uniform in the absence of CNAs and does not model copy number-neutral loss-of-heterozygosity. Therefore, it might falsely interpret these copy number-neutral events as copy number losses. BiTSC^2^ does not scale well to a large number of cells.^88^ It also requires SNVs and CNAs from the same set of cells as SCARLET,^56^ limiting its practical application because of the technical challenges of the single-cell whole-genome amplification processes.

To be more practical, SCsnvcna^87^ places SNVs on a CNA tree, where the SNVs and CNAs come from independent sets of cells. Since SNV and CNA cells from the same subclone are expected to exhibit similar cellular prevalence, SCsnvcna applies a Bayesian probabilistic model to combine genotype constraints on the tree and cellular prevalence to find the solution with the highest joint posterior probability. Given the observed genotypes for SNVs and the CNA tree, SCsnvcna uses MCMC sampling to search for the underlying genotype matrix and infer the placement of SNVs, SNV cells, and hyperparameters. It accounts for common single-cell sequencing errors, such as false positives, false negatives, and missing data, making it robust for datasets with high error rates. It also models the cell-sampling bias, with a normal distribution of the distance between the cellular prevalence of an SNV cell and a CNA cell. To model the SNV loss resulting from copy number loss and implement the Dollo model, SCsnvcna computes the distance between cellular prevalence only for nodes unaffected by mutation loss. However, despite its higher specificity than SCARLET, it has a lower sensitivity for detecting SNV loss.

In contrast to other methods, PACTION (PArsimonious Clone Tree integratION)^67^ integrates the existing clone trees of SNVs and CNAs and the corresponding clonal proportions to obtain a refined clone tree and a new clone proportion matrix, requiring minimum correction in the input matrices. In the refined clone tree, each node is a combined clone, and each edge represents mutation events that alter the parent clone to generate the child clone. In other words, it solves the PACTION problem for bulk DNA sequencing data using mixed-integer linear programming under the principle of parsimony. However, it does not consider the uncertainty in the input clone trees or quantify their effect on the solution space, which requires further improvement.

### Methods for building mutation trees

COMPASS (COpy number and Mutations Phylogeny from Amplicon Single-cell Sequencing)^88^ is currently the only method that infers a joint mutation tree of SNVs and CNAs using single-cell amplicon sequencing data. It computes the tree likelihood by marginalizing the attachments of cells to node genotypes, significantly enhancing computational efficiency and enabling the efficient processing of data from thousands of cells. It infers the tree with the maximum posterior probability via a simulated annealing algorithm with MCMC steps to avoid exhaustively searching the huge tree space. COMPASS models the observed reference and mutated read counts, the total read count in a region (gene), sequencing errors, allele-specific dropouts, and doublets, making it robust against amplicon-specific local coverage fluctuations. It may also model WGD by adding a single event. However, the number of CNAs is limited to at most one per lineage because of the challenges in accurately inferring copy numbers beyond three and detecting a loss preceded by a gain (or *vice versa*) in the same region from noisy sequencing data. Moreover, COMPASS also struggles to identify subclones characterized solely by CNAs, and its performance evaluation was limited to blood malignancies with Tapestri data.

## Phylogenetic inference methods integrating copy number alterations and structural variants

Only a few methods have been developed to integrate SVs for phylogenetic inference [Table 3], mainly because of challenges in the reliable detection, structural complexity, and phylogenetic interpretation of SVs.^76^ Because WGS is often required for SV detection, these methods are mostly applied to WGS data.

**Table 3:** Software tools available for cancer phylogenetics using CNAs and SVs.

Tool	Data	Model	Algorithm	Pros	Cons	Availability
**Methods without clonal decomposition**						
GRAFT	Allele-specific CN, SV, SNV multiplicity	ISM	ML	Infers phylogeny from complex SVs for bulk data	Requires robust data pre-analysis	https://www.sanger.ac.uk/tool/graft
**Methods with clonal decomposition**						
SubcloneSeeker	Variant cellular prevalence	ISM	Enumeration	Enumerates all possible subclone structures	Requires robust data pre-analysis	https://github.com/yiq/SubcloneSeeker
TUSV	Average CNs of segment and breakpoint	Dollo	MP	Demonstrates the feasibility of modeling SVs with CNAs	Applicable to a limited number of variants	https://github.com/CMUSchwartzLab/Sc-TUSV-ext
TUSV-ext	Average CNs of allele-specific Segment, breakpoint, and SNV	Dollo	MP	Integrates SNVs, CNAs, and SVs within a single framework for bulk data	Applicable to a limited number of variants	https://github.com/CMUSchwartzLab/TUSV-ext
*Sc-TUSV-ext*	Average CNs of allele-specific Segment, breakpoint, and SNV	Dollo	MP	Integrates SNVs, CNAs, and SVs within a single framework for single-cell data	Applicable to a limited number of variants	https://github.com/CMUSchwartzLab/Sc-TUSV-ext

The methods developed for single-cell data are shown in italics. CN: Copy number; CNA: Copy number alteration; GRAFT: Genomic rearrangement assembly for tumors; ISM: Infinite sites model; ML: Maximum likelihood; MP: Maximum parsimony; SNV: Single-nucleotide variant; SV: Structural variant.

### Methods without clonal decomposition

GRAFT (Genomic Rearrangement Assembly For Tumours)^96^ is the only method for inferring cancer phylogeny using SVs from bulk data without subclonal reconstruction. Assuming that each breakpoint occurs only once on a single chromosome, GRAFT determines the order of genome rearrangements and assembles genomic segments into digital karyotypes from SVs, allele-specific CNAs, and SNVs. It also reconstructs a rooted binary segmental evolution tree according to the inferred order of rearrangements and uses the ML method to estimate the timing of the rearrangements. Nonetheless, GRAFT requires complete datasets with well-curated mutations and may become less accurate using datasets with few mutations.

### Methods with clonal decomposition

SubcloneSeeker^97^ provides a unified framework for subclonal reconstruction from bulk data. It can accept many types of somatic variants, including SNVs, CNAs, and SVs, as long as their cellular prevalence can be calculated from either sequencing or microarray datasets. SubcloneSeeker supports the ISM by assuming that the same mutation cannot occur independently in two subclones that do not share a descendant relationship and that there are no back mutations. It enumerates all possible subclone structures consistent with the input data and reduces the solution space via additional linkage information, such as those inferred from a pair of primary and relapsed tumors from the same patient. SubcloneSeeker can generate a unique solution using multiple samples from both primary and relapsed tumors; however, its applicability is limited to datasets lacking linkage information.

TUSV^76^ is the first method that integrated SVs for the joint inference of clonal subpopulations and phylogenies from bulk data. It takes the SVs and CNAs that Weaver^98^ generated as input, which are converted into a mixed copy number of SV-supporting breakpoints and segments. TUSV assumes that breakpoints follow the ISM but allows violations of the ISM caused by allelic loss by considering the copy number of a lost allele as zero, which leads to the Dollo model. It also aims to minimize the deviation between the true and observed data while maintaining consistency between SVs and CNAs and the low evolutionary cost of the phylogenetic tree, which is solved using a coordinate descent algorithm.^68^ TUSV-ext^77^ and Sc-TUSV-ext^78^ are extensions of TUSV^76^ that incorporate SNVs into the clonal phylogenetic framework. TUSV-ext was designed for bulk data, whereas the Sc-TUSV-ext was tailored for single-cell data. Both methods allow allele-specific segmental copy numbers and joint phasing of breakpoints. They apply the Dollo model to both SNVs and breakpoints and improve the scalability of the algorithm by subsampling the full set of variants for phylogenetic inference to reduce redundant information. Moreover, Sc-TUSV-ext uses the MED distances computed by MEDICC2^36^ to cluster single cells into potential clones, upon which an algorithm similar to that used by TUSV-ext is applied for optimization. Both methods showed enhanced accuracy compared to previous methods that only considered subsets of variant types, suggesting new ways of integrating diverse forms of somatic mutations to obtain a more comprehensive understanding of cancer evolution.

## Application of phylogenetic inference methods using copy number alterations

Phylogenetic trees built using CNAs offer insights into the heterogeneity and evolutionary dynamics of genetic alterations during cancer initiation and progression and have been used to unravel ITH, decipher the molecular mechanisms of metastasis, understand treatment resistance, and inform early cancer detection [Table 4].

**Table 4:** Application of phylogenetic inference methods using CNAs in practice.

	Method	Cancer	Data type	Number of patients (or datasets)	Reference
Intratumor heterogeneity	Multiple (PhylogWGS)	Pan-cancer	WGS (bulk)	2658	99
	Custom	Esophageal	WGS (bulk and single-cell)	15	100
Metastasis	MEDICC	Melanoma	WES (bulk)	4	101
	CONIPHER	Lung	WES (bulk)	421	11,102
Treatment resistance	MP	Breast	WGS (single-cell)	8	13
	Sitka	Breast (PDX)	WGS (single-cell)	7	103
Early detection	MP	Colon	WES or WGS (bulk)	4	32
	PhylogicNDT	Multiple myeloma	WGS (single-cell)	24	104
	CNETML	Ovarian	WGS (bulk)	5	105

CNA: Copy number alteration; CNETML: Copy number evolutionary tree maximum likelihood; CONIPHER: Correcting noise in phylogenetic evaluation and reconstruction; MEDICC: Minimum event distance for intra-tumour copy-number comparisons; MP: Maximum parsimony; PhylogicNDT: Phylogic N-dimensional with timing; PDX: Patient-derived xenograft; WES: Whole exome sequencing; WGS: Whole-genome sequencing.

CNA-based phylogenetic trees have been used to reveal the patterns of clonal evolution, determine the timing of driver mutations, and identify early genetic alterations that predispose individuals to cancer. In the pan-cancer analysis of the whole genome initiative, 11 methods (including PhyloWGS^91^), some of which utilized CNAs, were used for subclonal reconstruction to characterize ITH from WGS data of 2658 patients across 38 cancer types. The results revealed the importance of ITH and its drivers, providing a comprehensive resource for annotated subclonal events.^99^ In another study,^100^ CNA trees of early esophageal adenocarcinoma and their precursor Barrett's esophagus lesions in 15 patients were reconstructed using a custom program based on breakpoint presence called from WGS data of bulk samples and single cells. These trees showed recurrent patterns, including early bi-allelic *TP53* inactivation, frequent association of high CNA burdens with WGD, copy number heterogeneity before aneuploidy, and independent malignant transformation.

CNA-based phylogenies derived from metastatic samples are critical for elucidating the mechanisms underlying metastasis. Using WES data from primary, metastatic, and ctDNA samples from four patients with metastatic melanoma, phylogenies reconstructed using MEDICC showed branches with potential WGDs, suggesting that WGD is not required to facilitate metastasis and that CNAs occur both before and after WGD.^101^ These phylogenies also revealed parallel or convergent evolution, in which CNAs occurred more than once in different parts of the tree. In the tracking cancer evolution through therapy (TRACERx) study of 1644 tumor samples from 421 patients with non-small cell lung cancer,^11^ the phylogenies of SNVs and CNAs were reconstructed using CONIPHER^71^ to determine the timing of somatic events. These phylogenetic trees revealed clonal and subclonal driver mutations and WGDs and contributed to evaluating the selection and timing of driver events in 401 tumors. Patients with tumors with recent subclonal expansions (shown on the terminal branches of the trees) had significantly shorter disease-free survival time. Overall, the results of the TRACERx study demonstrate the prognostic value of CNA-ITH, recent subclonal expansions, and subclonal WGDs. The trees were also used to assess the timing of divergence and modes of dissemination for 126 tumors with metastasis, showing that metastases diverged late in most cases and predominantly exhibited monoclonal dissemination.^102^ The seeding clones of metastases in the primary tumor were also analyzed to identify genomic events that confer metastatic potential. The results suggest that metastasizing clones show subclonal expansion and are larger than non-metastasizing clones, likely due to positive selection. Together with other analyses, phylogenetic inferences indicate the potential of evolutionary approaches for forecasting metastatic outcomes and treating emergent metastasizing clones in lung cancer.

When given pre- and post-treatment tumor samples, CNA-based trees can be used to follow treatment response and identify subclones likely to metastasize, relapse, or become resistant to therapy. For example, a study employed the classical MP method to reconstruct CNA-based phylogenies using single-cell data from eight patients with triple-negative breast cancer.^13^ The phylogenies generated confirmed that only diploid CNPs remained after neoadjuvant chemotherapy in four patients with clonal extinction. In contrast, adaptive selection enabled the expansion of preexisting clones and convergent evolution in the other four patients with clonal persistence. The pre-existence of chemoresistant clones suggests potential early diagnostic opportunities for detecting these clones before therapy to predict which patients will likely benefit from treatment. Sitka was used to reconstruct CNA trees from single-cell WGS data in a multiyear time-series study on breast epithelium and primary triple-negative breast cancer patient-derived xenografts (PDXs).^103^ These trees were post-processed to identify clonal populations whose abundances were tracked over time to infer their fitness. The results on epithelial cell lines indicate that *TP53* mutations generated a broad clonal fitness landscape where a large number of clones with distinct CNAs showed different fitness values, and the high-fitness clones exhibited increased aneuploidy and high-level amplifications of proto-oncogenes common in breast cancer. These results suggest that drug treatment led to the emergence of cisplatin-resistant clones from phylogenetic lineages with low fitness and the eradication of high-fitness lineages from treatment-naïve settings. This inversion of fitness was reversed during drug holidays, suggesting that there is a fitness cost in treatment resistance.

Applying CNA-based phylogenetic inferences to pre-malignant and early-stage disease samples can facilitate early cancer detection. In a study on the evolutionary history of human colitis-associated colorectal cancer, the classical MP method was used to build CNA-based trees from multi-region WES or shallow WGS data from four patients with colitis-associated colorectal cancer to learn the temporal dynamics of CNAs.^32^ The trees generated indicate patterns of punctuated evolution of CNAs from low-grade dysplasia (LGD) to high-grade dysplasia (HGD) and ongoing chromosomal instability in HGD and colorectal cancer. The rapid increase in CNAs from LGD to HGD indicated a narrow timeframe for the early detection of high-risk LGD lesions. In a study on the characterization of multiple myeloma (MM) using genome sequencing of circulating tumor cells, PhylogicNDT^93^ was used to reconstruct the evolutionary dynamics of MM in patients from WGS data, demonstrating the use of circulating tumor cells to track mutation acquisition, clonal dynamics, and treatment response over time in a minimally invasive manner.^104^ These reconstructions highlighted the timing of *KRAS* mutations and the expansion of subclones carrying driver mutations that confer a fitness advantage to tumor cells. The trees also revealed the emergence of a high-risk clone during treatment before any potential resistance developed. In conjunction with other analyses, this study suggests the feasibility of employing a minimally invasive liquid biopsy-based screening approach for the early detection and monitoring of MM. In another study on the genomic history of high-grade serous ovarian carcinoma, CNETML^59^ was used to reconstruct phylogenetic trees from the shallow WGS data of five patients, suggesting that CNAs present in secretory cells carrying a *TP53* mutation and serous tubal intraepithelial carcinomas were clonal.^105^ This finding supports the conclusion that the trajectory of high-grade serous ovarian carcinoma is likely to be determined at the earliest stages and highlights the importance of early cancer detection.

Despite the various applications of cancer phylogenetic inference methods using CNAs, they have mostly been used in retrospective studies. Given that many phylogenies are possible, researchers often need to use multiple methods to obtain consistent results.^3^ Moreover, the field still faces many challenges in clinical translation, owing to data complexity and difficulties in inference.^3^ As WGS enters mainstream clinical practice, cancer phylogenetics will play an increasingly more practical role in studying cancer mechanisms.^106^

Because the field of cancer phylogenetics is rapidly evolving with new computational methods, this study may not encompass all the latest developments. Some new methods being developed but not yet fully published are not included here. This includes SPICE, which reconstructs multiple equally plausible solutions for CNA evolution while accounting for complex CNAs and scaling to realistic numbers of samples.^107^ Additionally, methods based on array data are also not covered, such as TuMult,^35^ FISHtrees,^108^ and PISCA,^109^ although they may be applicable to DNA sequencing data.

## Conclusion

Cancer phylogenetic trees using CNAs are widely used to track and understand cancer evolution, providing insights into ITH and the complexities of cancer progression across many cancer types. These insights may be of direct clinical interest, as they can promote early prevention by identifying factors driving early tumorigenesis and guide patient treatment by identifying drug combinations or actionable mutations.^3^^,^^103^ Phylogenetic trees inferred from different patient cohorts may also help identify recurrent patterns important for cancer treatment.^11^ Thus, developing precise and efficient phylogenetic inference methods and their appropriate applications will make valuable contributions to the broader field of oncology research, ultimately benefiting patients with cancer.

Most methods assume that the input data are accurate, which is likely violated in practice owing to detection errors. Therefore, it is important to ensure that the input data are well-preprocessed and contain few errors. The accuracy of phylogenetic inferences from bulk data also depends on sequencing depth, the number of samples, and tumor purity.^45^ A higher sequencing depth allows for higher sensitivity in detecting rare subclones, whereas more samples and more differences between samples in their clonal composition increase accuracy and reduce ambiguity in phylogeny inference.^51^^,^^72^^,^^89^ Only a few available methods account for the noise and errors in the input data, most of which have been developed for single-cell data because of their low quality.^55^^,^^56^^,^^71^^,^^72^^,^^87^^,^^88^^,^^95^ With improvements in single-cell sequencing technologies, more information may be extracted from input data to enhance downstream analyses.

Different assumptions employed by the methods we presented may restrict their application to real data; therefore, caveats are needed to better understand the underlying models and interpret the results. Strong model misspecifications can result in inaccurate trees, emphasizing the need to test the robustness of different methods to violations of their assumptions.^5^ Different methods may generate conflicting conclusions from the same data; however, many studies have applied one specific method and have not assessed the robustness of the inferences to method changes.^4^ Hence, it is recommended that multiple analytical methods be used to obtain reliable results. As many trees may be consistent with bulk sequencing data from a few samples, caution is needed when drawing firm conclusions from a single tree.^51^

Despite the availability of the methods we presented, new phylogenetic inference methods that can better utilize CNAs and address issues related to scalability, data characteristics, and model complexity are still needed in the future for better evolutionary analysis of somatic cells to advance our understanding of cancer evolution. Currently, only a few methods are scalable for large datasets with more variants and clones because of their computational complexity,^36^^,^^55^^,^^80^^,^^88^ which calls for more efficient methods. Machine and deep learning approaches have enabled remarkable breakthroughs in many fields of computational biology and have also made progress in phylogenetics.^110^ Recent proof-of-concept studies have shown that machine learning methods can accelerate phylogenetic tree search algorithms^111^ or increase the accuracy and efficiency of branch length estimation.^112^ These advances hold promise for incorporating machine learning into phylogenetic inferences using CNAs. Samples taken at multiple time points over a long period allow researchers to better track the evolutionary trajectories of cancers driven by chromosomal instability; however, only a few methods have been developed that utilize temporal information.^59^^,^^113^^,^^114^ As liquid biopsies become more widespread, more longitudinal samples are expected to be collected, thus demanding better methods for utilizing temporal information. Although multiple-scale chromosomal changes are common in real data, only a few methods can handle them because of their inherent complexity.^36^ For example, no methods can handle complex SVs that affect multiple genomic regions simultaneously, such as chromothripsis. Because the integration of CNAs with other variants showed good performance, it is anticipated that there will be more sophisticated and realistic evolutionary models and computational methods that can capture the interplay among different somatic mutations, including complex SVs, gene expression, and epigenetic alterations.^115^

In summary, cancer phylogenetic inference methods using CNAs and other somatic variants detected from DNA sequencing data will undergo further improvement with the development of new sequencing techniques, more realistic models of evolution, and more advanced inference algorithms. To facilitate their applications in practice, these methods are required to be more user-friendly and more robust to noise in real data. Comprehensive benchmark studies are also needed to evaluate the performance of different methods. When WGS is fully integrated into clinical practice, patients may have their phylogenetic trees reconstructed in a short time from longitudinal monitoring or multi-region samples. These trees will reveal the unique spatiotemporal evolutionary dynamics of somatic mutations in each patient and assist the tailoring of treatment according to evolutionary principles.

## Authors contribution

Bingxin Lu: conceptualization, investigation, data curation, writing-original draft preparation, writing-reviewing, and editing. The author, Bingxin Lu, approved the final version of the manuscript.

## Ethics statement

None.

## Declaration of generative AI and AI-assisted technologies in the writing process

ChatGPT was used to improve the readability and language of the work. After using this tool, the author reviewed and edited the content as needed and takes full responsibility for the content of the publication.

## Funding

None.

## Data availability statement

All data are available within this manuscript.

## Conflict of interest

The authors declare that they have no known competing financial interests or personal relationships that could have appeared to influence the work reported in this paper.
